# Thermal Energy Storage in Concrete by Encapsulation of a Nano-Additivated Phase Change Material in Lightweight Aggregates

**DOI:** 10.3390/nano14141180

**Published:** 2024-07-11

**Authors:** Iván Carrillo-Berdugo, Juan Jesús Gallardo, Nazaret Ruiz-Marín, Violeta Guillén-Domínguez, Rodrigo Alcántara, Javier Navas, Juan Antonio Poce-Fatou

**Affiliations:** Departamento de Química Física, Facultad de Ciencias, Universidad de Cádiz, Puerto Real, 11510 Cádiz, Spain; jj.gallardo@uca.es (J.J.G.); nazaret.ruiz@uca.es (N.R.-M.); violeta.guillendo@alum.uca.es (V.G.-D.); rodrigo.alcantara@uca.es (R.A.); javier.navas@uca.es (J.N.)

**Keywords:** building materials, lightweight aggregates, nanomaterials, phase change materials, thermal comfort, thermal energy storage

## Abstract

This work discusses the applicability of lightweight aggregate-encapsulated *n*-octadecane with 1.0 wt.% of Cu nanoparticles, for enhanced thermal comfort in buildings by providing thermal energy storage functionality to no-fines concrete. A straightforward two-step procedure (impregnation and occlusion) for the encapsulation of the nano-additivated phase change material in lightweight aggregates is presented. Encapsulation efficiencies of 30–40% are achieved. Phase change behavior is consistent across cycles. Cu nanoparticles provide nucleation points for phase change and increase the rate of progression of phase change fronts due to the enhancement in the effective thermal conductivity of *n*-octadecane. The effective thermal conductivity of the composites remains like that of regular lightweight aggregates and can still fulfil thermal insulation requirements. The thermal response of no-fines concrete blocks prepared with these new aggregates is also studied. Under artificial sunlight, with a standard 1000 W·m^−2^ irradiance and AM1.5G filter, concrete samples with the epoxy-coated aggregate-encapsulated *n*-octadecane-based dispersion of Cu nanoparticles (with a phase change material content below 8% of the total concrete mass) can effectively maintain a significant 5 °C difference between irradiated and non-irradiated sides of the block for *ca.* 30 min.

## 1. Introduction

The use of thermal energy storage (TES) technologies in buildings could help smoothing temperature indoors and reducing the total energy consumption in buildings by storing and later releasing solar thermal energy. The applicability of TES in buildings has caught the interest of researchers during the last decade. Latent heat (related to phase changes) is recognized as the most attractive form of TES because it provides high-energy storage densities compared to sensible heat (related to temperature changes) or thermochemical heat (related to chemical reactions). The implementation of latent TES in buildings is approached by introducing encapsulated phase change materials (PCMs) into different building elements [1], such as bricks [2,3,4], concrete walls [2,5,6,7,8,9], wallboards [10,11,12] or plasters [13,14,15], among others [16,17,18]. There is, however, a dissonance between the research interest and the actual application of these TES technologies in buildings. Simó-Solsona et al. [19] identified that the economic, technical and contextual regulatory barriers that constrain the use of TES in buildings dissuade professionals from considering TES technologies as viable for building projects. The use of PCMs in buildings remains limited because central technical issues like efficiency, reliability and simplicity of implementation are yet to be addressed.

Cabeza et al. [20] published a detailed list of organic and inorganic PCMs whose physical properties justify their suitability for the enhancement of temperature comfort via TES in buildings. Suitability implies that the PCM satisfies, at least partially, a series of desirable characteristics, including phase change temperatures within the range of thermal comfort, high phase change enthalpy and high thermal conductivity. Paraffins (mixtures of saturated hydrocarbons with general formula C_n_H_2n+2_) in general, and *n*-octadecane (C_18_H_38_) in particular, are promising candidates due to their high phase change enthalpies, but their low thermal conductivity, especially in liquid phase, is a known drawback for their exploitation in applications that demand latent energy storage [21].

The rate of progression of the phase change fronts, as described by the classical Stefan problem [22,23], is proportional to *α*^1/2^, where *α* is the thermal diffusivity of the PCM defined as [24]

(1) $$\alpha =\kappa \cdot \rho^{-1}\cdot c^{-1}$$

which is a function of the thermal conductivity *κ*, the density *ρ* and the isobaric specific heat *c* of the PCM. Thus, low thermal conductivity values hinder phase change kinetics, which is the reason why a PCM tends to remain solidified at the edges and corners of macroencapsulation systems, from which heat transfer is not as effective as from an ideal constant temperature wall [25]. This limits the maximum achievable efficiency of thermal energy charge and discharge, but encapsulation or shape stabilization is still central for the application of any PCM in buildings because it confines the PCM and prevents its leakage once it has transitioned to liquid phase, so TES functionality is preserved and the mechanical integrity of the building is not compromised [26,27,28].

Alternatively, the relatively small dimensions of micrometric systems allow for phase change of the complete encapsulated or shape-stabilized volume [26,29]. This is why microencapsulation of PCMs has become a trending strategy for the incorporation of these materials into building elements. SiO_2_, for instance, is a recurring material for microencapsulation, typically achieved by hydrolysis of the tetraethyl orthosilicate precursor on the microemulsified PCM in water suspension [30,31,32]. There are other alternatives in which the PCM is encapsulated by shape-stabilizing in a microporous silica support, with similar encapsulation efficiencies [33]. Current research explores the possibility of incorporating and shape-stabilizing PCMs directly into cement-based materials [34,35]. The utilization of nanomaterials has also gained much attention recently [36]. These can be introduced as dispersed additives that increase the effective thermal conductivity of the PCM [37,38], or to fabricate ultraporous networks to be filled with a PCM [39,40,41,42].

The current picture summarizes the use of PCMs in buildings as an emerging application in an early stage of development, and basic research works on the matter are circumscribed in two lines of action, microencapsulation or shape-stabilization and addition of nanomaterials, aiming to compensate the efficiency and reliability problems of TES due to the low thermal conductivity of organic PCMs. The complexity of some microencapsulation procedures (for instance, the control of emulsion particle size [43] or the synthesis of nanomaterial-based supports) certainly does not contribute to simplifying the implementation. The addition of nanomaterials should be addressed with caution because increasing the effective thermal conductivity of the PCM is as important as not increasing the effective thermal conductivity of the overall composite, so that they remain useful as insulation materials as well.

Lightweight expanded clay aggregates (LECAs) are proposed as candidates for the purpose of shape-stabilization of PCMs. Encapsulation in their microporous structure should effectively provide the same benefits of microencapsulation and a more advantageous incorporation into buildings, as LECAs are used for concrete, mortar and grout in buildings and constructions of civil engineering. TES-functional aggregates could be used in precast concrete panels for cladding [44], no-fines concrete slabs as an air-permeable layer between two cavities in dynamic insulation walls [45], or even no-fines concretes for other vertical applications (now that sand scarcity appears to be an emerging issue with major environmental implications) [46] with an adequate structural reinforcement and hydrophobic treatment [47]. This work reports the preparation of leak-free TES-functional aggregates by impregnation of LECAs with *n*-octadecane, with and without Cu nanoparticles in suspension, and occlusion with epoxy resin. *n*-Octadecane exhibits melting temperatures around 28 °C, thus being suitable for thermal control by latent heat storage in buildings at regions with average monthly temperatures well above 22 °C during warm seasons. The addition of Cu is motivated by its notable thermal conductivity, low market price and immediate availability. The characterization of their phase change enthalpy and temperature, thermal conductivity and specific heat is undertaken, as well as their incorporation into no-fines concrete blocks, whose thermal response is also characterized. The discussion is approached from applied and fundamental perspectives. The work presents an easily operated two-step procedure for the encapsulation of the PCM in LECAs, and a laboratory-scale method for the characterization of their thermal response in concrete samples that can be easily run with a solar simulator.

## 2. Materials and Methods

### 2.1. Preparation of Aggregate-Encapsulated PCM Samples

**Figure 1:**
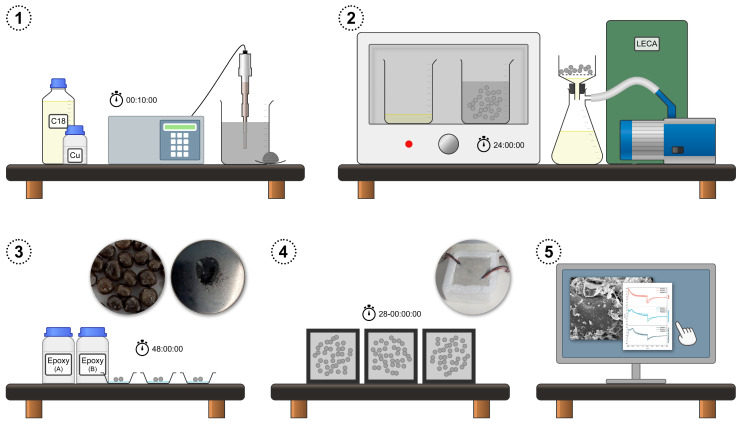
Summary of the experimental procedure for aggregate-encapsulated PCM samples: (1) dispersion, (2) impregnation, (3) occlusion, (4) concrete preparation and (5) thermal response characterization.

*n*-Octadecane (C18, Sigma-Aldrich, St. Louis, MO, USA, 99%, melting point 26–29 °C) was forced to permeate into the porous structure of the LECAs (Weber Saint-Gobain, Paris, France, Arlita Leca dur, 2–10 mm granulometry, density 350 kg·m^−3^, <34% water absorption) by immersion in the liquid PCM for 24 h in a vacuum oven (Goldbrunn Therm, Munich, Germany, Goldbrunn 450) at 60 °C. After that, PCM-impregnated aggregates were collected, and the excess of liquid *n*-octadecane was drained. Residual *n*-octadecane on the surface of the aggregates was allowed to run down and drain for 30 min at 60 °C. Therefore, the PCM content in the aggregates is determined by capillarity in the porous structure. The aggregates were then coated by epoxy resin (Eurotex, Seville, Spain) to occlude the structure and prevent leakage of the PCM. The excess of epoxy resin was drained as well, and the composite was then left to cure for 48 h at room conditions. For the incorporation of Cu nanoparticles (Sigma-Aldrich, ≥99.5%, 60–80 nm particle size), the required amount for a mass fraction of 1.0 wt.% was dispersed in *n*-octadecane by liquid phase ultrasonication (Elma, Singen, Germany, Elmasonic P30H, 80 kHz) for 10 min, before the aggregates were immersed. Thus, two types of epoxy-coated aggregate-encapsulated PCM samples were characterized: without Cu nanoparticles (C18/LECA/Epoxy) and with a 1.0 wt.% load of Cu nanoparticles homogeneously dispersed in the PCM (Cu@C18/LECA/Epoxy). Figure 1 summarizes the preparation procedure.

### 2.2. Characterization of the Microstructure, Composition, and Thermal Characteristics

Because these samples are at an early stage of development, their characterization focuses on the microstructure, composition, and thermal characteristics of the sample. Mechanical properties should be characterized in future works, after all thermal characteristics have been measured and the functional value of a given sample type has been determined to be valid for application.

Scanning electron microscopy (SEM, FEI, Nova NanoSEM-450, Hillsboro, OR, USA, operated at 5 kV acceleration voltage) combined with energy dispersive X-ray (EDX) spectroscopy (EDAX, Octane Ultra silicon drift detector, 100 mm^2^, Mahwah, NJ, USA) were used to determine the presence of *n*-octadecane and Cu nanoparticles at the core of the porous structure of the aggregate. Samples for imaging were embedded in a matrix of a synthetic phenol-formaldehyde polymeric resin (Struers, Copenhagen, Denmark, PolyFast, black bakelite hot mounting resin with carbon filler, thermosetting) and cut in half so their core was exposed. Each sample type was prepared in duplicate for SEM imaging.

The phase change enthalpy and temperature and specific heat were measured by differential scanning calorimetry [48] (DSC, Netzsch, Selb, Germany, DSC 214 Polyma, concave Al pan, pierced lid). Temperature, heat flow and Tau-R calibrations were performed prior to sample analysis, using a standard calibration procedure on In, Sn, Zn, Bi and CsCl certificated reference samples. The instrument precision was determined to be better than ±2.0% for the measurement of enthalpy and specific heat. Because aggregates cannot be contained in the DSC pans, samples were prepared as described in Section 2.1 but using disk-like aggregate fragments (*ca.* 4 mm diameter, 1 mm thickness) prepared with a hole punch cutter. Each sample type was prepared in duplicate for DSC measurements. The temperature program for the study of phase change behavior runs a cycle with (i) a heating step from 0 °C to 50 °C at 1 °C·min^−1^, (ii) an isothermal step for 5 min and (iii) a cooling step from 50 °C to 0 °C at 1 °C·min^−1^. A faster cycle that runs (iv) a heating step from 0 °C to 50 °C at 5 °C·min^−1^ and (v) a cooling step from 50 °C to 0 °C at 5 °C·min^−1^ was iterated nine times between each characterization cycle. Only the DSC signal registered at 1 °C·min^−1^ (cycles 1, 11, 21, 31, 41 and 51) was considered for characterization purposes. Thermal stability analyses based on ~50 thermal cycles are not unprecedented [49]. This type of analysis should not be based on a large, arbitrary number of cycles, but on the changes in the DSC curve descriptors (i.e., on-set temperature, FWHM, enthalpy, etc.). This scheme allows evaluating the sample performance over many cycles in a significantly reduced time frame. The temperature program for the determination of specific heat runs a temperature-modulated step from 0 °C to 20 °C and from 30 °C to 50 °C at 1 °C·min^−1^, with an amplitude of ±1 °C and a period of 120 s. Specific heat measurements were performed in triplicate for each sample.

Thermal conductivity was measured by the transient hot-bridge technique (THB, Linseis, THB-100, hot-point sensor type C, 10 mW input power, 10 s measurement time). The instrument precision was determined to be better than ±3.5% for the measurement of thermal conductivity. The THB technique with the hot-point sensor requires the aggregates and their composites to be mechanically ground into powder. Each sample type was prepared in duplicate for THB measurements. Thermal conductivity measurements were performed at room temperature and repeated at least 10 times for each sample.

### 2.3. Testing of the Thermal Response under Artificial Sunlight

No-fines concrete blocks were prepared with Portland limestone cement (Adeo, Ronchin, France, Axton cement type CEM II/B-LL, complies with UNE-EN 197-1:2011 [50]) and either LECA, C18/LECA/Epoxy composites or Cu@C18/LECA/Epoxy composites as coarse aggregates were evaluated and compared. A 0.4 water-to-cement mass ratio was used, aiming for the minimum necessary water content as per supplier recommendation for completing hydration reactions during hardening. The aggregate-to-cement mass ratio was 0.1 for LECA, 0.16 for C18/LECA/Epoxy and 0.15 for Cu@C18/LECA/Epoxy. This low aggregate-to-cement ratio responds to the need of flat concrete surfaces to avoid influence of significant rugosity on the testing under solar irradiation, but it is not suitable for immediate application. The concrete mixture was added and left to harden in a tetragonal template (42 mm × 42 mm × 15 mm) for 48 h and cured for a month at room temperature under air atmosphere before starting characterization procedures. Average sample compositions after curing are detailed in Table 1.

Two sets of three samples were prepared. The first set was intended for fracture and imaging of the concrete microstructure under an optical microscope (Nikon, SMZ800, microscope, Tokyo, Japan; Volpi, Intralux 4100, halogen light source, Auburn, AL, USA; uEye, UI-2230-C, CCD camera, Obersulm, Germany). The second set of samples was tested under sunlight from a solar simulator (Abet Technologies, Sun 3000, xenon lamp, standard AM1.5G filter, Milford, CT, USA) configured for the irradiance on the squared facets of the samples to be 1000 W·m^−2^. The temperature of the irradiated and the non-irradiated sides of the sample was measured using Pt100 resistance thermometers (Labfacility, DM-503, 5 mm × 2 mm ceramic support, Bognor Regis, UK, −50 °C to 550 °C temperature measurement range, class A tolerance). The tolerance of the sensor at the maximum working temperature is below ±0.24 °C. The temperature values were directly recorded using analogical channels in a data acquisition card (Measurement Computing, USB-TEMP-AI, Norton, MA, USA), and monitored over time using a non-commercial software program developed by the authors. Each temperature value was the average over 100 replicas, with a waiting time of 200 ms between each value. Samples were framed in expanded polystyrene to minimize heat loss due to convection at the non-irradiated sides during the tests. In a typical test, the sample is first allowed to equilibrate its temperature with the environment, and then irradiated with the solar simulator for 2 h. After that, irradiation is ceased, and the sample is allowed to equilibrate its temperature with the environment again. This test was performed in triplicate for each sample.

## 3. Results and Discussion

### 3.1. Core Microstructure and Composition of Aggregate-Encapsulated PCM Samples

**Figure 2:**
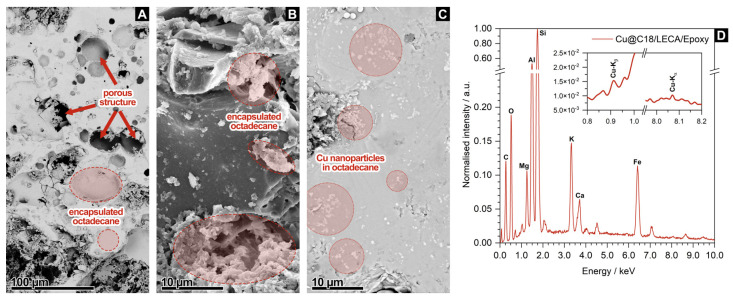
Representative SEM images of (**A**) a C18/LECA/Epoxy aggregate sample and (**B**,**C**) a Cu@C18/LECA/Epoxy aggregate sample, together with (**D**) the EDX spectrum of the latter.

This first section discusses the validity of the proposed method for the preparation of C18/LECA/Epoxy and Cu@C18/LECA/Epoxy composites before proceeding with the characterization of the phase change behavior and thermal properties. SEM imaging and EDX spectroscopy were used to determine the presence of *n*-octadecane and Cu nanoparticles at the core of the aggregates. Figure 2A shows a representative SEM image of a C18/LECA/Epoxy sample, in which it is easy to recognize that the porous structure of the aggregate is partially filled with *n*-octadecane. Figure 2B shows a representative SEM image of a Cu@C18/LECA/Epoxy sample, in which *n*-octadecane appears to be adhered to the edges and sides of the porous structure. Figure 2C displays a region of this sample that is particularly saturated with *n*-octadecane, in which contrast differences highlight the presence of submicron structures that are recognized to be Cu nanoparticles.

To further confirm the presence of Cu nanoparticles at the core of the aggregate, Figure 2D plots the EDX spectra acquired for a Cu@C18/LECA/Epoxy sample. The analysis is based on K_α_ and K_β_ X-ray emissions [51]. EDX identifies the presence of Si (*K*_α_ = 1.739 keV), Al (*K*_α_ = 1.486 keV), Mg (*K*_α_ = 1.253 keV) and O (*K*_α_ = 0.525 keV), which are expected, as clays are typically a mixture of SiO_2_, Al_2_O_3_ and MgO, Fe (*K*_α_ = 6.398 keV), which is a frequent substitute of Al and Mg, and K (*K*_α_ = 3.312 keV) and Ca (*K*_α_ = 3.690 keV), from K_2_O and CaO species that are also part of its composition [52]. There is also C (*K*_α_ = 0.277 keV), with a quantification of 23.70 wt.%, which is sufficiently high to consider it is not only due to adventitious species and to confirm the presence of *n*-octadecane at the core of the aggregate. EDX also identifies the presence of Cu (*K*_α_ = 8.040 keV and *K*_β_ = 0.930 keV), with a quantification of 0.94 wt.%, which confirms the presence of Cu nanoparticles at the core of the aggregates, in a concentration that matches the mass fraction of Cu that was originally dispersed in *n*-octadecane. All the above provides sufficient evidence to validate the proposed method for the preparation of C18/LECA/Epoxy and Cu@C18/LECA/Epoxy composites.

### 3.2. Phase Change and Thermal Properties of Aggregate-Encapsulated PCM Samples

**Figure 3:**
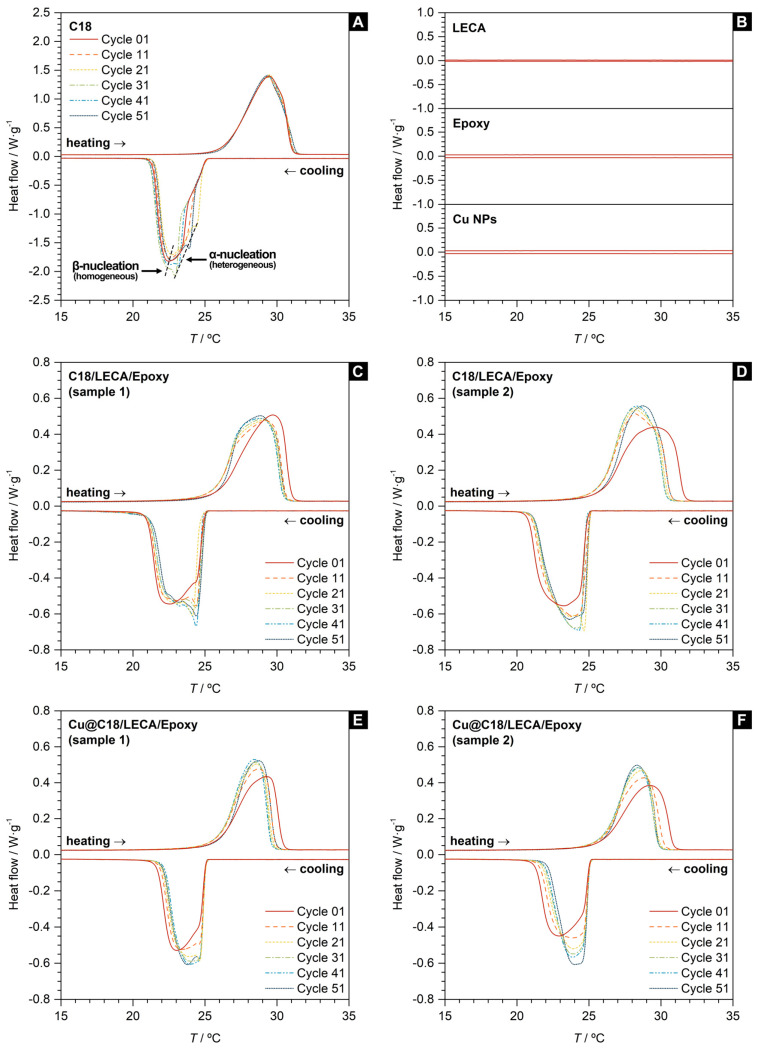
DSC curves for (**A**) *n*-octadecane, (**B**) LECA, Epoxy resin and Cu nanoparticles, (**C**,**D**) C18/LECA/Epoxy aggregate samples and (**E**,**F**) Cu@C18/LECA/Epoxy aggregate samples. Curves are referenced to heating and cooling rates of 1 °C·min^−1^.

Figure 3 showcases the DSC curves from the consecutive DSC cycles performed on the relevant aggregate-encapsulated PCM samples, and Table 2 summarizes the thermodynamic indicators that characterize their phase change behavior. Figure 3A presents the DSC curves for bulk *n*-octadecane. The thermodynamics of the melting and crystallization processes are identical, with enthalpies of Δ*H*_m_ = 239.3 ± 0.4 J·g^−1^ and Δ*H*_c_ = −240.9 ± 0.5 J·g^−1^, but they exhibit asymmetric kinetics, as proven by the single-peak and double-peak shape of their respective heat flow signals. The crystallization of *n*-octadecane proceeds via heterogeneous and homogeneous nucleation [30], with the formation of α- and β-crystals at peak temperatures of *T*_c,α_ = 23.7 ± 0.7 °C and *T*_c,β_ = 22.5 ± 0.1 °C. The temperature at which heterogenous nucleation occurs varies between cycles, as it is indicated by the standard deviation. The extrapolated onset temperatures (which are recommended to describe the starting point of the phase transition because they are relatively independent of parameters like heating or cooling rates) are found to be *T*_m_ = 26.1 ± 0.1 °C and *T*_c_ = 25.2 ± 0.1 °C, which sets a thermal hysteresis, ΔT* = *T*_m_ − *T*_c_, of 0.9 ± 0.2 °C for *n*-octadecane.

All these results are in good agreement with those reviewed in the literature [53], which proves the goodness of the DSC measurements and justifies the selection of *n*-octadecane as a PCM to provide LECAs with TES functionality because, compared to other organic PCMs [20], it has a high phase change enthalpy, onset temperatures within the range of thermal comfort for habitable buildings, and low thermal hysteresis.

Figure 3C,D present the curves for C18/LECA/Epoxy samples. The phase change enthalpy of these composites is proportional to the encapsulated loading of *n*-octadecane in its porous core only. Figure 3B proves that no other component is susceptible to phase change within the range of temperatures under study. The encapsulation efficiency, *EE*, is a descriptor that quantifies the TES functionality of aggregates filled with a PCM [30], and it is calculated as

(2) $$EE=\frac{{\left(\Delta H_{m}+\Delta H_{c}\right)}_{\mathrm{encapsulated}}}{{\left(\Delta H_{m}+\Delta H_{c}\right)}_{\mathrm{bulk}}}\cdot 100$$

The C18/LECA/Epoxy samples under study have *EEs* over 40%, meaning nearly half the mass of these composites stores latent heat. The *EE* also represents an indirect determination of the PCM loading via calorimetry. The fact that this indicator is in good agreement with the direct determination via gravimetry further validates the proposed procedure for the preparation of these TES-functional composites.

Compared to bulk *n*-octadecane, melting and crystallization kinetics of encapsulated *n*-octadecane remain asymmetrical, and both processes preserve their starting temperatures and low thermal hysteresis. There is a remarkable difference for the peak temperature for heterogeneous nucleation, which shifts towards higher values and becomes much less variable between different cycles (*T*_c,α_ = 23.7 ± 0.7 °C for bulk *n*-octadecane, *T*_c,α_ = 24.5 ± 0.1 °C for encapsulated *n*-octadecane). This finding suggests that heterogeneous crystallization is no longer dependent on random nucleation events, as the porous structure at the core of the aggregates provides nucleation sites from which the phase change front progresses, thus becoming more repetitive between cycles.

Figure 3E,F present the curves for Cu@C18/LECA/Epoxy samples. These samples have *EEs* over 30%. The *EEs* of these samples are noticeably lower than those of C18/LECA/Epoxy samples, which suggests that the addition of Cu nanoparticles hinders the impregnation of the porous structure and limits the PCM loading into the aggregate. The dispersion of Cu nanoparticles seems to have a significant impact on the crystallization of *n*-octadecane, as the peaks in the DSC cooling signal of Cu@C18/LECA/Epoxy samples are almost completely overlapped over cycles, with an associated peak temperature *T*_c_ = 24.2 ± 0.1 °C. This suggests the heterogeneous (α) nucleation stage in the crystallization process is more dominant in Cu@C18/LECA/Epoxy samples than it is in either bulk *n*-octadecane or C18/LECA/Epoxy samples. Cu nanoparticles serve as nucleation sites dispersed throughout the volume of *n*-octadecane, in contrast to the randomly distributed nucleation sites in bulk *n*-octadecane or the nucleation sites provided by the porous structure of the aggregates, which would be located at the boundaries of that volume only. Such interpretation is consistent with existing evidence from molecular dynamics, previously reported by Liu and Rao [54], indicating that Cu surfaces assist the crystallization of this alkane.

**Figure 4:**
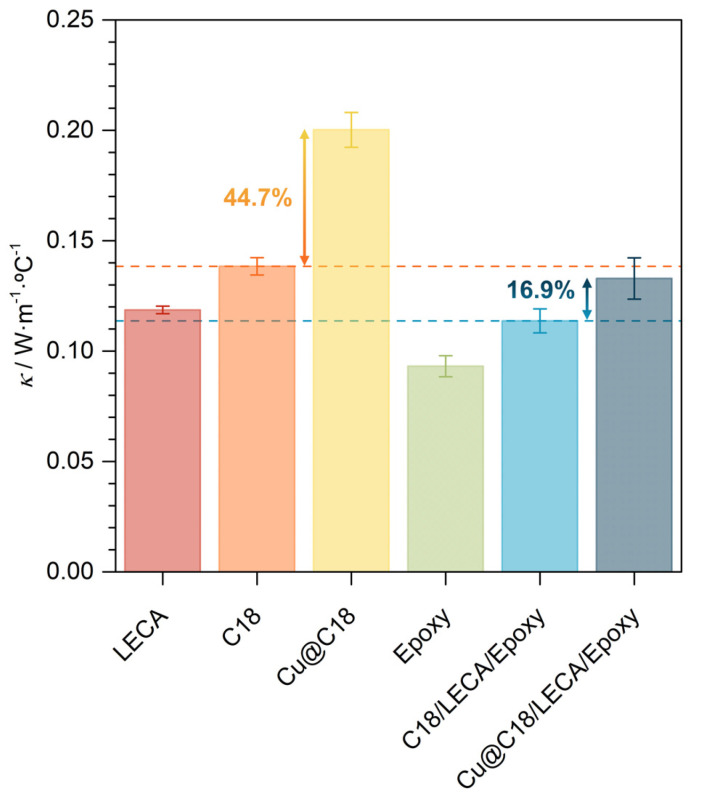
THB-acquired thermal conductivity values, at 30 °C, for LECA, pure *n*-octadecane (liquid), *n*-octadecane with 1.0 wt.% of Cu nanoparticles (liquid), epoxy resin and C18/LECA/Epoxy and Cu@C18/LECA/Epoxy samples.

**Figure 5:**
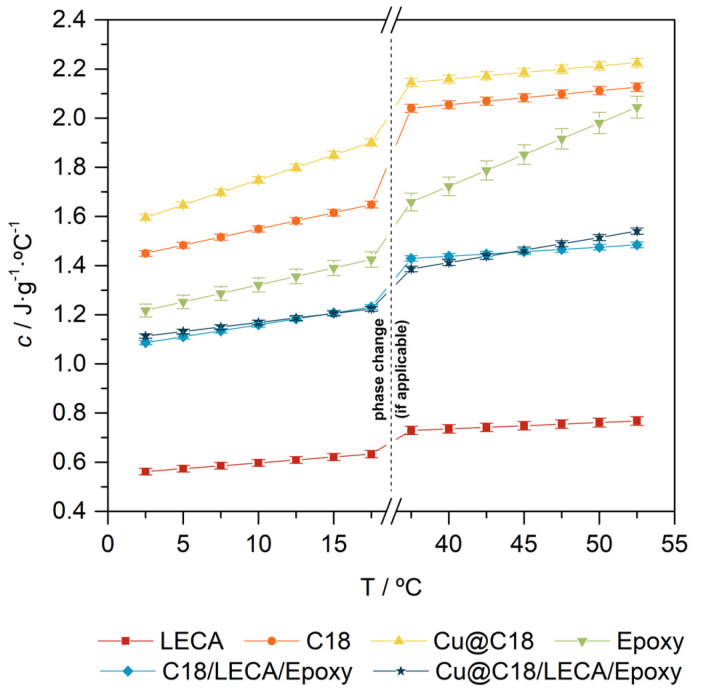
DSC-acquired specific heat values, as a function of temperature, for LECA, pure *n*-octadecane, *n*-octadecane with 1.0 wt.% of Cu nanoparticles, epoxy resin and C18/LECA/Epoxy and Cu@C18/LECA/Epoxy samples.

Regardless of the nature of the nucleation process, the progression of the phase change front is expected to be boosted by the increase in the effective thermal conductivity of *n*-octadecane with Cu nanoparticles. The change in this thermal property of the PCM can ease the thermal charge and discharge processes of the TES-functional aggregate, and thus the storage and release of latent thermal energy. For this statement to be valid, it should be verified that, in relation to thermal diffusivity, the increase in thermal conductivity is not compensated by an increase in specific heat. For that, thermal conductivity and specific heat measurements were carried out. The measured values for these two physical properties for all the samples under characterization are presented and compared in Figure 4 and Figure 5. As a general observation, the results for bulk *n*-octadecane, in either solid or liquid state, are in excellent agreement with those reviewed in the literature [53].

It is verified in Figure 4 that the addition of 1.0 wt.% of Cu nanoparticles increases the thermal conductivity of *n*-octadecane by 44.7%. According to effective medium approach-based models for the thermal conductivity of mixtures, the effective thermal conductivity of the dispersion is much lower than expected for the given mass fraction of Cu nanoparticles (*ca.* 150 W·m^−1^·°C^−1^ for a particle size of 60–80 nm) [55], which suggests a significant effect of the solid–liquid interfacial thermal resistance [56]. Still, an enhancement is determined. The effective thermal conductivity of Cu@C18/LECA/Epoxy is higher, by 16.9%, than that of C18/LECA/Epoxy, and higher, by 12.0%, than that of LECA without any additives. These findings are significant for the application because they indicate the effect of Cu nanoparticles is localized at the core of the aggregates, in which phase change occurs and high thermal conductivity is required for reliable latent heat storage and release, but the overall thermal conductivity of the composite is still sufficiently low to ensure thermal insulation in concrete.

On the other hand, the specific heat of *n*-octadecane is increased by an average 12.8% in solid phase and 4.9% in liquid phase by dispersion of 1.0 wt.% of Cu nanoparticles. Although the specific heat of bulk Cu (0.385 kJ·kg^−1^·°C^−1^) [24] is lower than that of *n*-octadecane, and so should be that of the dispersion, the specific heat of Cu nanoparticles is higher compared to its bulk because the contribution from surface atom vibrations becomes much more significant as particle size decreases [57]. The enhancement is significant, but sufficiently low to safely conclude that the increase in thermal conductivity is not compensated by an increase in specific heat, meaning that thermal diffusivity increases, and so do the melting and crystallization rates. On the other hand, the specific heat of Cu@C18/LECA/Epoxy is practically the same as that of C18/LECA/Epoxy, and differences between them are undistinguishable from the uncertainty of the measurement.

### 3.3. Thermal Response of Concrete with Aggregate-Encapsulated PCM

The potential applicability of aggregate-encapsulated *n*-octadecane for TES in concrete is sufficiently justified given the characterized phase change behavior and thermal properties. This section goes through an observation of the resulting structures and interfaces upon incorporation of the composites in concrete and a direct determination of their thermal response under solar irradiation.

**Figure 6:**
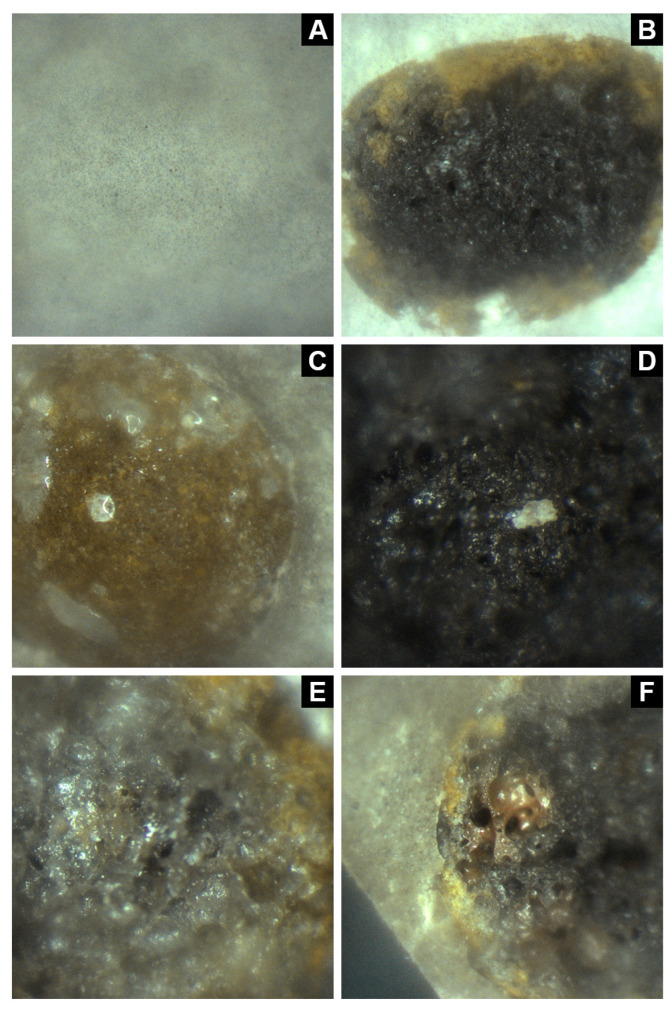
Optical microscopy images of the microstructure of no-fines concrete blocks with (**A**,**B**) regular LECA, (**C**,**D**) with C18/LECA/Epoxy composites and (**E**,**F**) with Cu@C18/LECA/Epoxy composites.

Two sets of three no-fines concrete samples were prepared, each with a different aggregate type: regular LECA, C18/LECA/Epoxy composites or Cu@C18/LECA/Epoxy composites. The first set was fractured, and the exposed microstructures were observed via optical microscopy. Figure 6A,B show, for reference purposes, the smooth structure of the hardened no-fines cementitious matrix, and the porous core of a regular aggregate embedded in the former. Figure 6C displays an image of a C18/LECA/Epoxy composite and confirms that epoxy shells remain intact after cement hardening, thus effectively encapsulating *n*-octadecane at the core of the porous aggregate, as it is observed in Figure 6D. Figure 6E,F show images of the core of Cu@C18/LECA/Epoxy composites, which are also saturated with the nano-additivated *n*-octadecane dispersion. Another key observation is that no discontinuities are observed across the aggregate–matrix interface in any case.

**Figure 7:**
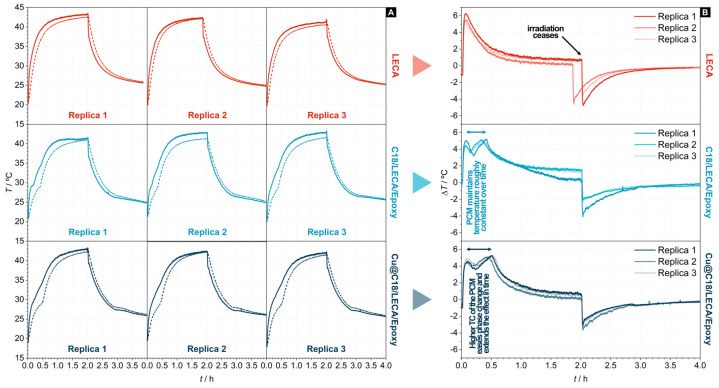
Thermal response vs. time of concrete samples with LECA, C18/LECA/Epoxy composites or Cu@C18/LECA/Epoxy composites under artificial sunlight irradiation. In (**A**), solid and dashed lines represent *T*_top_ (irradiated side) and *T*_bottom_ (non-irradiated side), respectively. In (**B**), solid lines represent the temperature difference (Δ*T* = *T*_top_ − *T*_bottom_) for each replica and sample type.

Figure 7A plots the measured temperatures at the irradiated (*T*_top_) and non-irradiated (*T*_bottom_) sides of the relevant samples as a function of the time under irradiation. The consistency of the measured *T* vs. *t* curves across multiple replicas for each sample proves the reproducibility of the procedure and the robustness of the set-up. The temperature difference (Δ*T* = *T*_top_ − *T*_bottom_) in Figure 7B provides a more comprehensive visualization and eases the analysis of the transient thermal performance of these samples. Δ*T* vs. *t* curves convey that concrete samples with regular LECA cannot maintain a significant temperature difference between the irradiated and the non-irradiated sides, whereas those filled with *n*-octadecane are able to “trap” heat and effectively maintain it. The effect of phase change appears in these curves in the form of roughly constant temperature segments in the heating phase of the test, before irradiation ceases. Concrete samples with C18/LECA/Epoxy can maintain a significant 5 °C difference for *ca.* 20 min, whereas those with Cu@C18/LECA/Epoxy can do so for *ca.* 30 min. In view of the basic characterization that was accomplished in previous sections of this paper, and that no sources of bias are affecting this result (because both samples have the same dimensions, the same *n*-octadecane content, the same starting temperature at both sides, and were exposed to the same irradiance), it is fair to conclude that the better performance of concrete with Cu@C18/LECA/Epoxy is due to the increased effective thermal conductivity of the PCM with Cu nanoparticles, and the effect of such increment on the progression of phase change fronts.

## 4. Conclusions

The two-step procedure (impregnation and occlusion) for the encapsulation of an *n*-octadecane-based dispersion of Cu nanoparticles (1.0 wt.%) in lightweight expanded clay aggregates was proven successful. It ensures a leak-free, shape-stabilization of the phase change material without any penalty for the simplicity of its implementation, as it is scalable and straightforward in terms of execution. The resulting composites have heat storage capacities within 70–80 J·g^−1^, which competes well with other alternatives for the incorporation of phase change materials in building materials, such as those presented by Yu et al. [34] (encapsulation of *n*-octadecane by impregnation in a red mud-based geopolymer hollow microsphere and precipitation of CaCO_3_ on its surface) or Topçu et al. [35] (shape-stabilized mixture of myristic acid and lauric acid in a sepiolite porous support), with heat storage capacities around 49.5 J·g^−1^ and 78.8 J·g^−1^, respectively. The encapsulation efficiency was determined to be better than 30% in all cases here studied, and stable across consecutive cycles. The repeatability of the α-nucleation process during the crystallization of encapsulated *n*-octadecane was found to improve with respect to bulk *n*-octadecane, as the porous support provides many nucleation points at the boundary of the encapsulated volume. Even more nucleation points are available at the middle of the encapsulated volume of *n*-octadecane with the dispersion of Cu nanoparticles, in which case the heterogeneous (α) nucleation stage becomes dominant. The rate of progression of the phase change front is expected to be higher due to a significant increase, by 44.6%, of the effective thermal conductivity of *n*-octadecane with Cu nanoparticles, that is not compensated by an increase, by up to 12.8%, in its effective specific heat. The overall thermal conductivity of Cu@C18/LECA/Epoxy composites is higher than that of lightweight aggregates without any additives by 12.0% only. This indicates the effect of Cu nanoparticles is locally significant at the core of the encapsulation, and that the overall thermal conductivity of the composites is still sufficiently low to ensure thermal insulation if used as coarse aggregates in concrete. For all that, it is concluded that Cu@C18/LECA/Epoxy composites are very promising candidates to provide concrete with TES functionality. Such functionality was tested by subjecting no-fines concrete blocks with these aggregates to solar irradiation. Under artificial sunlight, with a standard 1000 W·m^−2^ irradiance and AM1.5G filter, concrete samples with regular lightweight aggregates cannot maintain a significant temperature difference between the irradiated and the non-irradiated sides, whereas concrete samples with C18/LECA/Epoxy and Cu@C18/LECA/Epoxy composites, with a phase change material content below 8% of the total concrete mass, can maintain a significant 5 °C difference for *ca.* 20 min and 30 min, respectively. This finding means that the inclusion of these new aggregates can improve the thermal response of concrete-based elements, which positively contributes towards temperature damping and thermal comfort.

## Figures and Tables

**Table 1:** Composition of no-fines concrete block-like samples with different aggregate types, and composition breakdown for each aggregate type. Mass fractions are referenced to the total dry mass of the block after the curing process. (*) Includes the mass of Cu nanoparticles in suspension.

Aggregate Type	Cement	Aggregate	LECA	PCM	Epoxy
LECA only	90.0 wt.%	10.0 wt.%	10.0 wt.%	0.0 wt.%	0.0 wt.%
C18/LECA/Epoxy	84.0 wt.%	16.0 wt.%	7.7 wt.%	7.5 wt.%	0.7 wt.%
Cu@C18/LECA/Epoxy	84.8 wt.%	15.2 wt.%	7.0 wt.%	7.5 wt.% *	0.7 wt.%

**Table 2:** Summary of the relevant indicators of the phase change behavior of bulk and encapsulated *n*-octadecane samples. Indicators are referenced to heating and cooling rates of 1 °C·min^−1^.

Sample	PCM wt.	Δ*H*_m_/J·g^−1^	−Δ*H*_c_/J·g^−1^	*EE*	*T*_m_/°C	*T*_c_/°C	ΔT*/°C	*T*_c,α_/°C	*T*_c,β_/°C
Bulk C18	--	239.3 ± 0.4	240.9 ± 0.5	--	26.2 ± 0.1	25.2 ± 0.1	1.0 ± 0.1	23.7 ± 0.7	22.5 ± 0.1
C18/LECA/Epoxy	38.9%	98.8 ± 0.2	98.1 ± 0.2	41.0%	25.8 ± 0.2	25.1 ± 0.1	0.7 ± 0.2	24.5 ± 0.1	22.9 ± 0.2
	43.8%	105.4 ± 0.7	105.4 ± 0.6	43.9%	25.8 ± 0.2	25.2 ± 0.1	0.6 ± 0.2	24.5 ± 0.1	23.6 ± 0.2
Cu@C18/LECA/Epoxy	33.2%	80.1 ± 0.3	77.9 ± 0.4	32.7%	25.6 ± 0.1	25.2 ± 0.1	0.4 ± 0.1	24.2 ± 0.1	
	30.3%	74.2 ± 0.2	73.7 ± 0.2	30.8%	25.9 ± 0.1	25.2 ± 0.1	0.7 ± 0.1	24.2 ± 0.1	

## Data Availability

Data will be made available on request.
